# The Best reviewers of International Brazilian Journal of Urology in 2020

**DOI:** 10.1590/S1677-5538.IBJU.2021.01.02

**Published:** 2020-11-18

**Affiliations:** 1 Universidade do Estado de Rio de Janeiro Unidade de Pesquisa Urogenital Rio de JaneiroRJ Brasil Unidade de Pesquisa Urogenital - Universidade do Estado de Rio de Janeiro - Uerj, Rio de Janeiro, RJ, Brasil; 2 Hospital Federal da Lagoa Rio de JaneiroRJ Brasil Serviço de Urologia, Hospital Federal da Lagoa, Rio de Janeiro, RJ, Brasil

## COMMENT

The peer review system is the soul of the Scientific journals. This process became an institutionalized part of the scholarly process in the latter half of the twentieth century (1). The serious review process is done by experts on the topic studied and is completely free depending on the goodwill and talent of the reviewers. The process of peer review is hard but improve the quality of published scientific manuscripts (2).

In 2020 the International Brazilian Journal of Urology received more than 900 papers and the reviewers were very important to the entire process of our Journal. As a Editor-in-Chief I would like to thanks all the reviewers and specially the Doctors: Alexandre Danilovic, MD (Hospital das Clínicas da Faculdade de Medicina da USP); Ralf Anding, MD (University Hospital Basel); John Denstedt, MD, PhD (Western University Canada); Trushar Patel, MD (University of South Florida) and Gustavo Ruschi Bechara, MD (Hospital Universitário Cassiano Antônio Moraes) who reviewed more than 5 articles during the year and strictly within the deadline.

Thanks a lot!!!!!

Luciano A. Favorito

Editor-in-Chief

International Brazilian Journal of Urology

**Figure:**
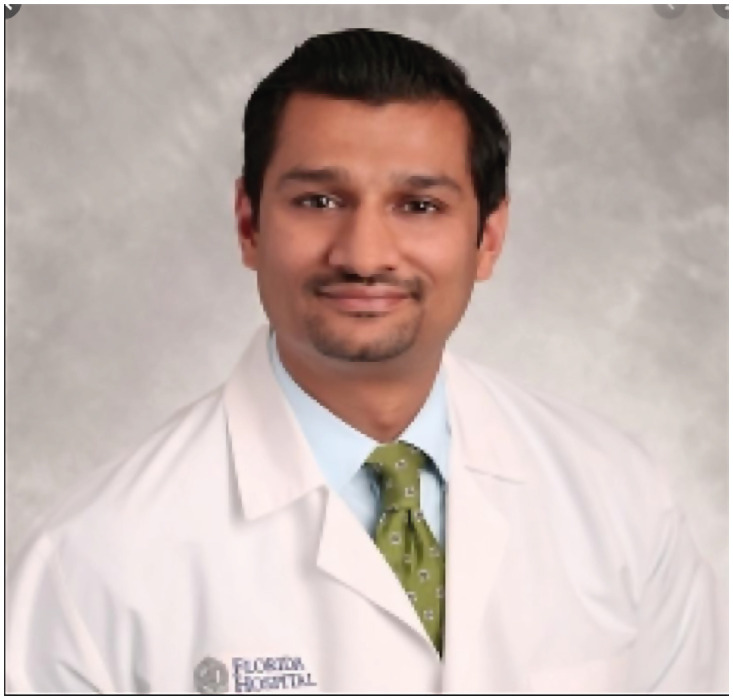
**Trushar Patel, MD**

University of South Florida

**Figure:**
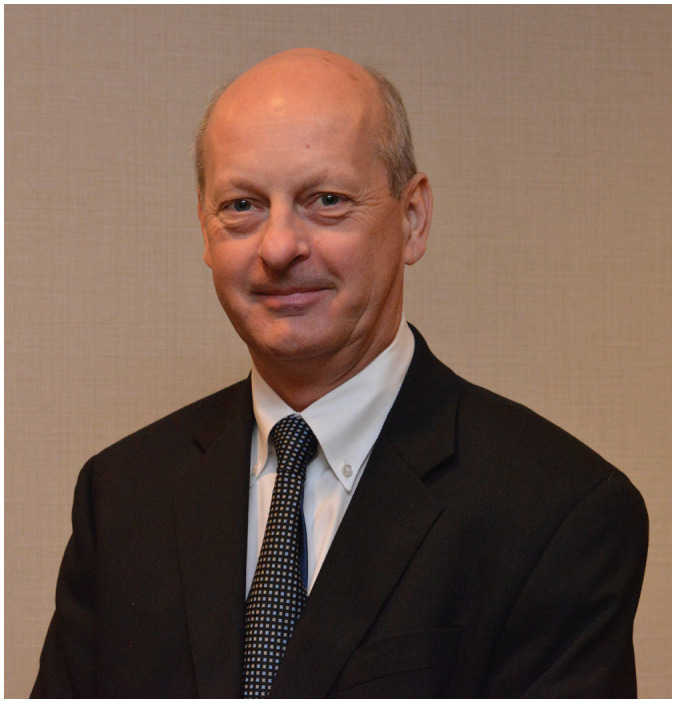
**John Denstedt, MD, PhD**

Western University Canad

**Figure:**
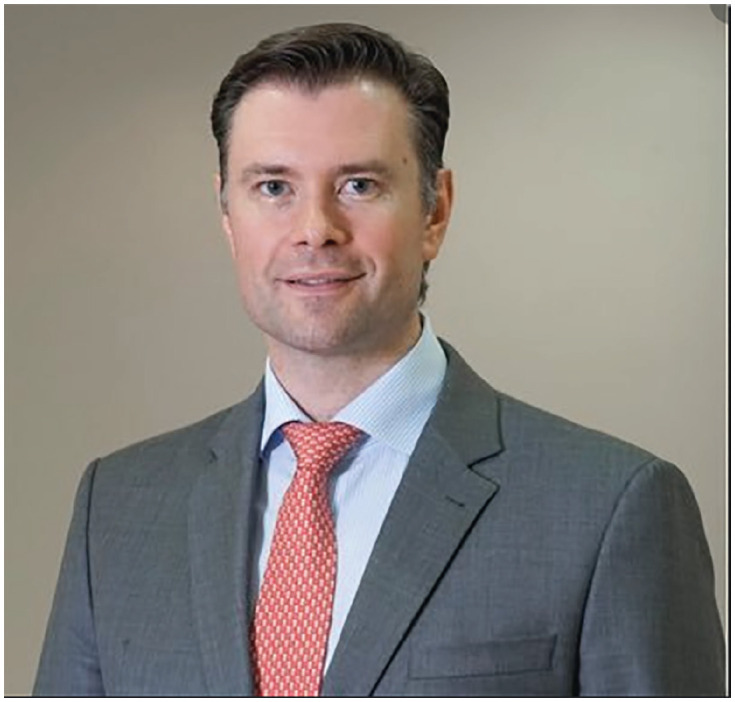
**Alexandre Danilovic, MD**

Hospital das Clínicas da Faculdade de Medicina da USP

**Figure:**
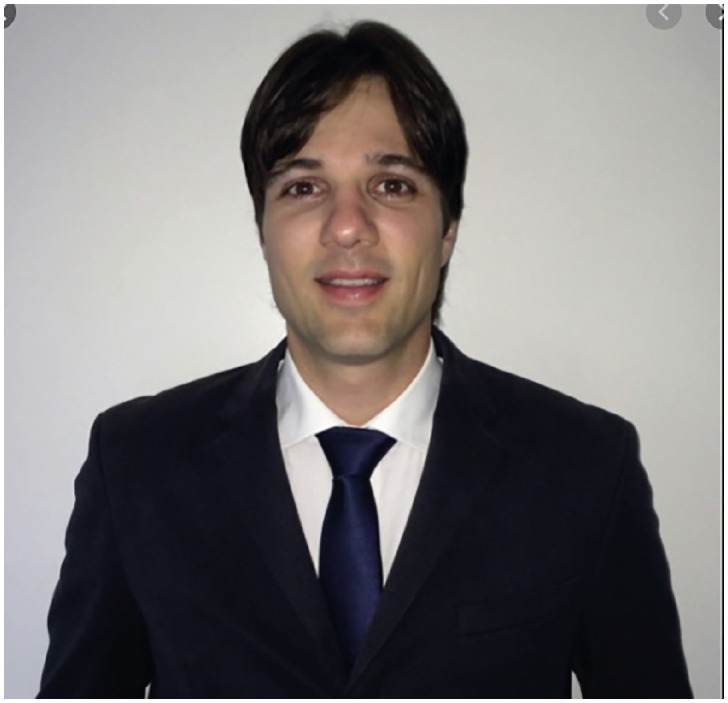
**Gustavo Ruschi Bechara, MD, PhD**

Hospital Universitário Cassiano Antônio Moraes

**Figure:**
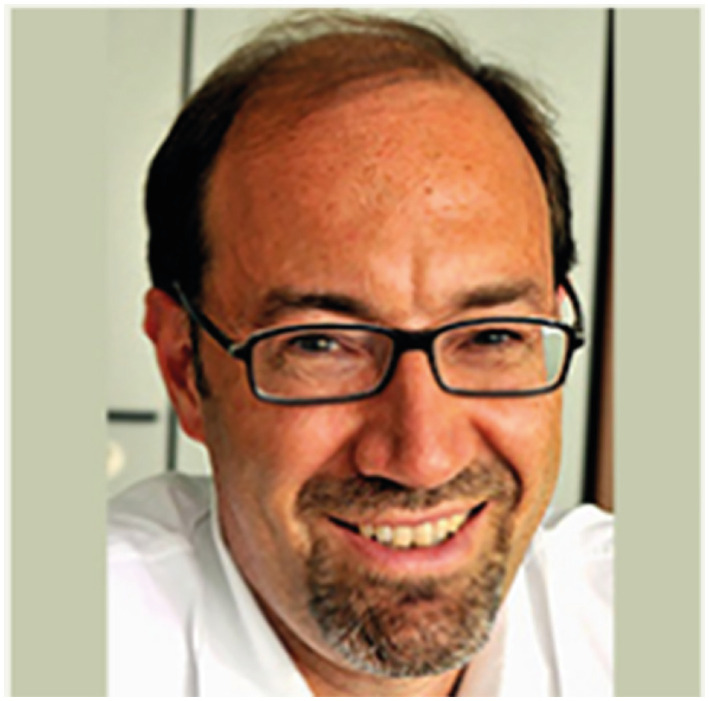
**Ralf Anding, MD**

University Hospital Basel
